# A novel wireless brain stimulation device for long-term use in freely moving mice

**DOI:** 10.1038/s41598-019-42910-7

**Published:** 2019-04-23

**Authors:** Melanie Alpaugh, Martine Saint-Pierre, Marilyn Dubois, Benoit Aubé, Dany Arsenault, Jasna Kriz, Antonio Cicchetti, Francesca Cicchetti

**Affiliations:** 1 Centre de Recherche du CHU de Québec (CHUQ), Axe Neurosciences, 2705, Boulevard Laurier, Québec, QC Canada; 2 CERVO Brain Research Center, Québec, QC Canada; 3 Département de Psychiatrie et Neurosciences, Université Laval, Québec, QC Canada

**Keywords:** Cellular neuroscience, Parkinson's disease, Microglia

## Abstract

Deep brain stimulation (DBS) has been used in clinical settings for many years despite a paucity of knowledge related to the anatomical and functional substrates that lead to benefits and/or side-effects in various disease contexts. In order to maximize the potential of this approach in humans, a better understanding of its mechanisms of action is absolutely necessary. However, the existing micro-stimulators available for pre-clinical models, are limited by the lack of relevant small size devices. This absence prevents sustained chronic stimulation and real time monitoring of animals during stimulation, parameters that are critical for comparison to clinical findings. We therefore sought to develop and refine a novel small wireless micro-stimulator as a means by which to study consequent behavioural to molecular changes in experimental animals. Building on previous work from our group, we refined our implantable micro-stimulator prototype, to be easily combined with intravital 2-photon imaging. Using our prototype we were able to replicate the well described clinical benefits on motor impairment in a mouse model of Parkinson’s disease in addition to capturing microglia dynamics live during stimulation. We believe this new device represents a useful tool for performing pre-clinical studies as well as dissecting brain circuitry and function.

## Introduction

We sought to create and refine a novel deep brain stimulator to study consequent molecular, thru to behavioural changes, in experimental animals. A number of different micro-stimulator devices have been previously developed. These stimulators differ in weight, size, stimulation parameters, ability to deliver chronic stimulation and the presence of an external power source. The majority of devices designed for rodents require external power sources to generate current^1^, which generally means that animals cannot simultaneously undergo stimulation and behavioural analysis. Some devices overcome this limitation by using tethers, magnetic waves or light sources to drive stimulation. However, all of these solutions have drawbacks since tethers can impact the movement and behaviour of mice, magnetic wave stimulators are limited to testing environments that can be contained within the required chambers, and light stimulation can only occur during the light phase of the light-dark cycle^2^. These constraints have led to the production of a light-weight wireless micro-stimulator for rats^2–4^. No such device had previously been developed for mice as their smaller size increases the difficulty of the task. However, such a device would have broad applicability to the study of neurological conditions as there are far more genetic mouse models of disease than there are rat models.

This breadth of applications is a critical component of the micro-stimulator as the potential benefits of DBS are currently being evaluated in at least twelve different neurological and psychiatric conditions^5^. Tangible outcomes for patients could be significantly accelerated by the development of better tools which accurately mimic clinical conditions in pre-clinical models. In this paper, we present a step-by-step description of the development of the first wireless implantable micro-stimulator that can deliver chronic stimulation in freely moving mice over several weeks and which allows for behavioral analysis. This device includes improvements on several fronts, such as better coating methods to optimize long-term performance, as well as enhanced surgical techniques to facilitate sustained portability of the stimulator. In addition, we have designed the prototype so that it can be coupled to 2-photon intravital imaging which permits the study of cellular responses to brain stimulation with high spatial and temporal resolution.

## Results

### Development of the first compact and adaptable micro-stimulator for sustained long-term brain stimulation in mice

**Figure 1:**
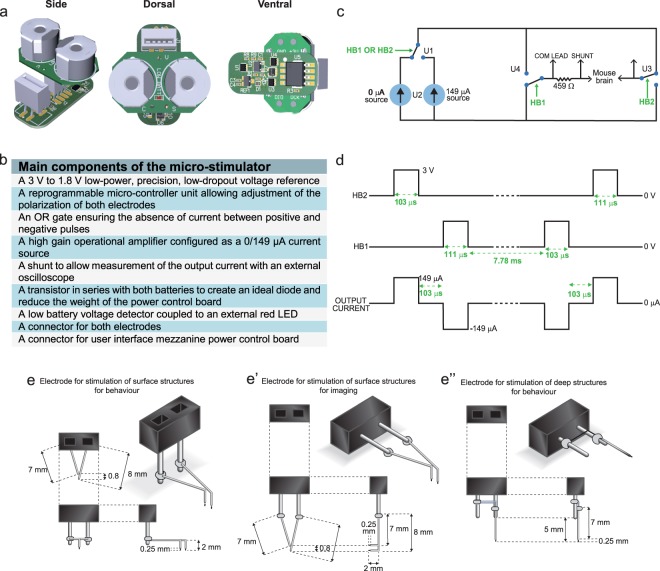
Micro-stimulator components, circuit and electrodes. (**a**) Simplified schematic of the micro-circuit board and battery support. (**b**) List of main components of the micro-stimulator. (**c**) Timing diagram demonstrating the offset between HB1 and HB2 as well as the summed output current. (**d**) Pulse duration, frequency and dead time (between positive and negative current pulses) of the stimulation paradigm. (**e-e”**) Types of electrodes designed for the various experimental applications: stimulation alone (**e**), short-term 2-photon imaging with stimulation of deep cerebral structures (**e’**) or long-term stimulation combined to repeated 2-photon imaging sessions (**e”**). Abbreviations: HB1: H bridge 1; HB2: H bridge 2; µA: microamperes; Ω: ohms, V: volts; LED: light emitting diode.

In order to perform chronic DBS, a light-weight stimulator capable of being carried on the back of the mouse for extended periods of time without hampering movement was designed. To achieve this, we developed an implantable micro-stimulator equipped with external battery cases and a red LED that permits experimenters to monitor and change the batteries without the requirement of a surgical procedure. Monitoring of the electrical output during the experiment is also possible through the use of specially designed external ports present on the battery board (Fig. 1a,b). Additionally, this stimulator retains the parameters of the device described in Arsenault *et al*.^1^, which critically allows for balanced positive and negative stimulation settings which have been demonstrated to generate the desired effect on local field potentials. The final circuit of this novel micro-stimulator is presented in Fig. 1c. Each circuit is equipped with a micro-controller unit that can be adjusted to alter the output program of the device even after implantation in a mouse. For our purposes, the program used resulted in a balanced alternating voltage (Fig. 1d). To increase the applications of this new system, a number of different electrodes were designed (Fig. 1e-e”). Bipolar electrodes were utilized for stimulation of cortical structures and were created with the connector in two different orientations, vertical and horizontal. A vertical alignment of the connectors was used for behavioural studies (Fig. 1e-e”) while a horizontal alignment was used for 2-photon imaging (Fig. 1e’). Monopolar electrodes were designed for stimulation of deeper brain structures, as this reduces tissue damage during implantation, and were utilized for behavioural analysis after stimulation of the subthalamic nucleus (Fig. 1e”).

### Coating methodology to prevent infiltration of fluid into micro-stimulators

**Figure 2:**
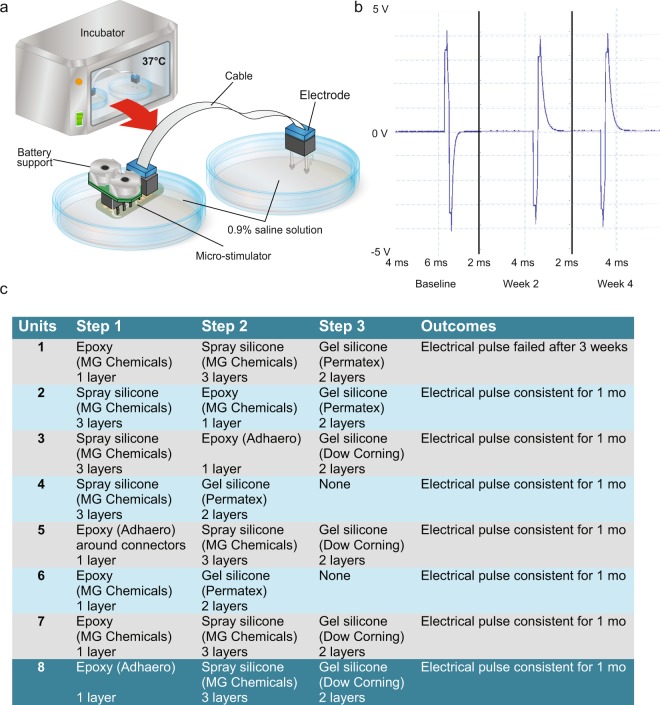
Prototype testing in *in vitro* conditions. (**a**) Schematic rendering of the *in vitro* set-up designed to test the long-term stability of the electrical output of the micro-stimulators after exposure to a saline solution. (**b**) A representative trace of a voltage reading from a micro-stimulator which successfully maintained a consistent electrical output for 1 month. (**c**) Summary of the various conditions/apparatuses tested with the selected condition (condition 8) highlighted in blue. Abbreviations: mo: month; V: volts.

Previous attempts at chronic stimulation in our laboratory had led to the observation that short-circuits could be rapidly provoked by infiltration of physiological fluids. To circumvent this problem, multiple methods of coating the device were tested in an *in vitro* paradigm designed to mimic the *in vivo* environment. The goal was to find a bio-compatible compound that is impervious to moisture. To conduct these tests, the micro-stimulator was placed in a petri dish containing 0.9% sodium chloride solution and was connected by a cable to the electrode which was maintained in a second saline-containing petri dish (Fig. 2a). Both petri dishes were covered in parafilm to prevent evaporation and to mimic the layer of skin that would cover the micro-stimulator components *in vivo*. The connectors, cables and battery board (with batteries) were all located outside the parafilm cover. The entire assembly of items was maintained in a 37 °C incubator for 4 weeks and the voltage output was monitored throughout this period (Fig. 2b). Several coatings were identified that could prevent the infiltration of fluid and permit the proper functioning of the unit for one month (units 2–8 Fig. 2c). Of the successful coating methods, unit 8 was selected as the Adhero brand epoxy was easier to apply and yielded more consistent coating than other tested compounds. Additionally, the outer Dow corning gel silicone layer has been previously reported to be biocompatible in similar applications^6,7^.

### Stable positioning of the micro-stimulator after optimization of surgical procedures

**Figure 3:**
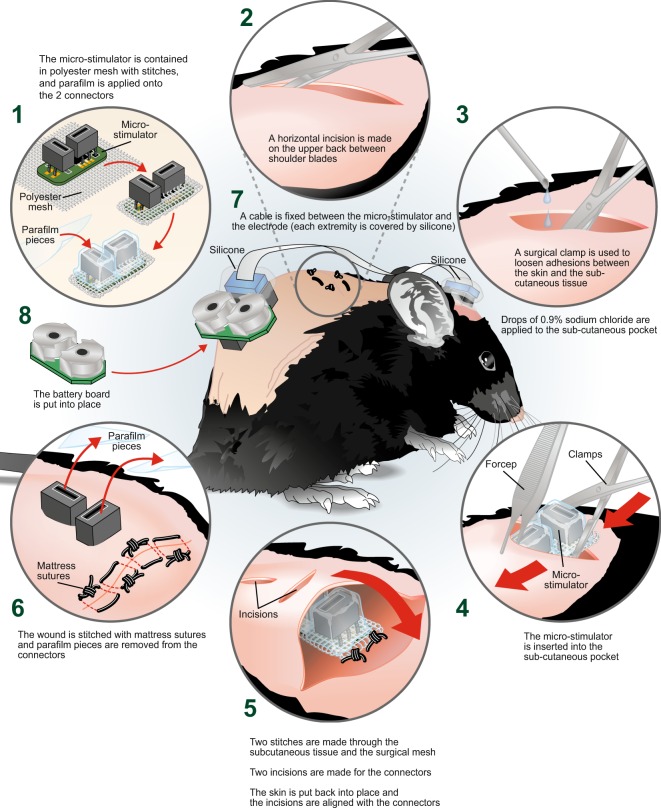
Surgical development of micro-stimulator implantation. Diagram of the various aspects of the surgical procedure optimized to prevent movement of the micro-stimulator and to facilitate long-term experimentation in freely moving mice. (**1**) The micro-stimulator was wrapped in polyester mesh and held in place with stitches. Parafilm was applied to the connectors to prevent fluid infiltration during implantation. (**2**) A small horizontal incision was performed on the upper back of the animal between the shoulder blades using surgical scissors. (**3**) A surgical clamp was used to loosen the connection between the skin and sub-cutaneous tissue. A few drops of 0.9% sodium chloride were added into the sub-cutaneous pocket. (**4**) The micro-stimulator was then inserted into the sub-cutaneous pocket using forceps and surgical clamps. (**5**) Sutures were used to bind the surgical mesh to the top and bottom of the subcutaneous tissue and two incisions were made above the connectors through which they were inserted. (**6**) The wound was closed using mattress sutures and the protective parafilm covering was removed from the connectors. (**7**) The cable was fixed between the micro-stimulator and the electrode (each extremity covered by silicone) and (**8**) the battery board was mounted into place.

Another challenge that we encountered was the tendency for the micro-stimulator to shift subcutaneously after surgical implantation, which tended to cause local skin irritation. To overcome this, we adapted a protocol that has previously been described for the insertion of cochlear implants in rats and mice^8–10^. This protocol involved wrapping the micro-stimulator in a polyester mesh prior to implanting it under the skin. This mesh increased tissue regrowth and facilitated integration of the device into the surrounding tissue which, consequently, reduced movement. Sub-cutaneous sutures were chosen to further abrogate movement of the device. During implantation, the connectors were covered in parafilm to ensure that no debris entered the connectors as this could interfere with the long-term functioning of the device. The parafilm was removed after successful implantation (see Fig. 3 for summary). While this protocol greatly improved the tolerability of the device, some mice still had occasional skin irritation after implantation, which could, however, be easily addressed by administration of green clay to problem areas.

### Combinatorial stimulation and intravital imaging

**Figure 4:**
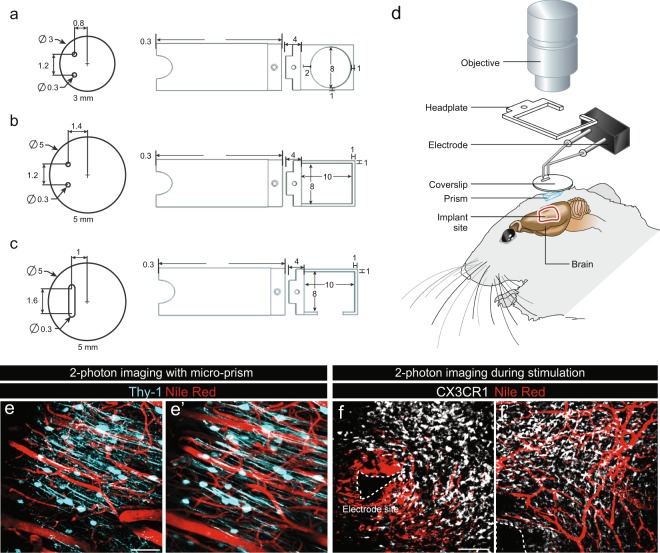
Concomitant 2-photon imaging and brain stimulation. (**a**–**c**) Three phases of design of the coverslips and head plates tested during the optimization of the intravital imaging protocol with the selected design illustrated in (**c**). (**d**) Schematic of the imaging set-up that was developed to overcome physical challenges such as the size of the objective, cranial window and electrode positioning. (**e**,**f**) Representative images of intravital 2-photon imaging in mice expressing CFP under control of the Thy-1 promoter (**e**-**e’**) and GFP under the control of the CX3CR1 promotor (**f**-**f’**) allowing identification of neurons and microglial cells respectively. (**e**-**e’**) Representative images of intravital 2-photon imaging of neurons using microprisms in the absence of stimulation and of (**f**-**f**’) microglia 2 weeks after implantation of the electrodes. In both conditions, blood vessels were visualized by injection of Nile red into the orbital vein of mice just prior to imaging. The electrode tip is delineated by a dotted line. Mice were implanted with electrodes 2 weeks prior to the commencement of imaging. Scale bars (**e-e**) = 50 µm, (**f-f’**) = 100 µm.

Since DBS has already demonstrated success in clinical settings, application of this technology to small animals would offer significant advantages in terms of better defining the molecular and cellular mechanisms involved in the previously described behavioural benefits. Coupling imaging with DBS could contribute greatly towards this goal. We therefore performed multiple optimizations to facilitate concomitant 2-photon intravital imaging and DBS. We used mice expressing GFP under the control of the CX3CR1 promotor that were additionally injected with Dextran-Texas-Red to visualize blood vessels. Several challenges were encountered during the development of this technique including respiratory movements from the animal, the necessity of incorporating both the electrode and the coverslip as well as the large size of the objectives that are required for 2-photon imaging. To minimize motion artifacts, a head plate was secured to the skull during the surgical procedure at the site of cranial window and electrode placement. Glass coverslips were modified to accommodate insertion of both electrodes. Multiple variations of both coverslips and head plates were attempted (Fig. 4a–c; with c being the selected model). Together, these adjustments overcame the first two obstacles, however, the reduction of the imaging area by the electrode remained a major problem. To address this, a micro-prism was attached to the base of the coverslip implanted in the cranial window, as previously described^11^. When combined with the aforementioned optimizations, a final protocol was established (Fig. 4d), which successfully permitted static imaging of the area proximal to the electrode (Fig. 4e,f) as well as in regions more distal to the electrode (Fig. 4f’). Using this approach, we were also able to record videos of microglial activity during stimulation (Supplementary Video 1).

### Applicability of the micro-stimulator to animal studies

**Figure 5:**
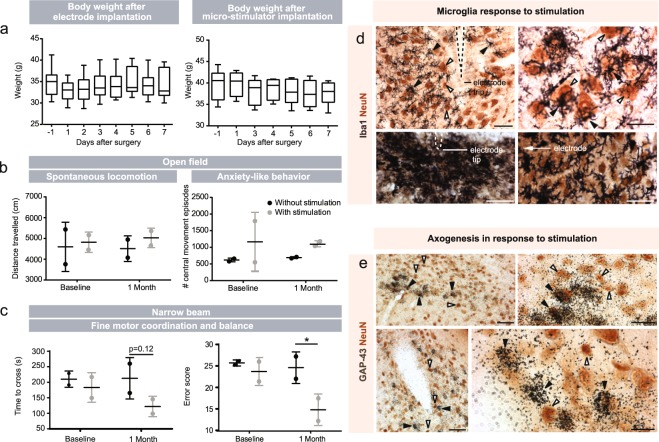
Health, behavioral and post-mortem assessment of experimental mice. (**a**) Weight fluctuation in mice after electrode and micro-stimulator implantation. No significant changes were detected. Statistical analyses were performed using repeated measures One-Way ANOVA followed by Dunnett’s multiple comparisons. N = 17 after electrode surgery and n = 8 after micro-stimulator surgery. (**b**,**c**) To test applicability of the micro-stimulator to behavioral parameters, we also performed the flex field (**b**) and narrow beam (**c**) tests in mice overexpressing human αsyn under the Thy-1 promoter (Thy1-αsyn), a model of Parkinson’s disease, before and after surgical implantation of the micro-stimulator and electrodes. N = 2 without stimulation, n = 2 with stimulation. Data is expressed as mean ± SEM. Statistics were performed by Two-Way ANOVA followed by uncorrected Fisher’s LSD test. (**d**) Double immunohistochemistry of microglia using Iba1 (in black) and NeuN (in brown). (**d**) Microglia are indicated by black arrowheads and neurons are indicated with white arrowheads. (**e**) Immunohistochemical labelling of neurons with NeuN using DAB (in brown) and *in situ* hybridization of GAP-43+ cells (silver grains). Neurons labeled only by NeuN are indicated with white arrows and neurons double-labeled with GAP-43 and NeuN are indicated with black arrowheads. Scale bars (**d,e**) = 50 μm. *p < 0.05.

Two important measures of success of the device are the long-term functionality and tolerability in mice and its ability to exert an effect on cells and cellular processes. We performed three different pilot studies to evaluate these critical measures of design success. In the first pilot study, we implanted electrodes and stimulators into WT mice and closely monitored the animals for more than a week after each surgical procedure. While in some instances skin irritation was seen, the mice demonstrated no other adverse effects to the presence of the stimulator. Food consumption was not altered, as indicated by the stability of the weight after electrode and micro-stimulator implantation. Mice did show a slight weight loss (~5%) 7 days after the micro-stimulator was added, however, this returned to normal at 9 days post-surgery (Fig. 5a).

As this study confirmed the tolerability of the procedure, we next implanted the micro-stimulator system into a small cohort of the Parkinson’s disease Thy1 α-syn mouse model and performed motor testing before and after one month of the subthalamic nucleus stimulation. Mice were still receiving stimulation when the second set of behavioural tests was performed. Although the small number of animals in this study limits the reliability of statistical analysis, groups were compared by two-way ANOVA and a significant reduction in the number of errors on the narrow beam was detected after 28 days of stimulation. While interesting, the most important observation is that we were able to determine that the Thy1 α-syn mice can perform motor tasks while wearing the micro-stimulator system, without signs of stimulation-associated behavioural deterioration. This is supported by the similarity between the distance travelled in the open field at baseline and 1-month time points (Fig. 5b) and the improved motor performance after chronic stimulation in the narrow beam test (Fig. 5c).

In the third pilot study, we assessed the effect of stimulation on specific cellular populations. Following stimulation, activation of microglia (Fig. 5d) and induction of GAP-43, a protein associated with axonal growth (Fig. 5e), were observed. Microglial activation occurred, as expected, in response to the implantation of the electrode, however, they were also responsive to chronic stimulation. After 24 h of stimulation (Fig. 5d upper panels), microglia were less activated than they were after 25 days of chronic stimulation (Fig. 5d lower panels). GAP-43 induction was seen as early as 3 days after stimulation, remarkably in the hemisphere contralateral to the implantation site, suggesting that brain stimulation has wide spread effects.

## Discussion

To circumvent a major problem in the use of DBS in small experimental animals, one of the main focuses of our design was to make our micro-stimulator light-weight by optimizing the circuits to achieve a total external weight of 4.7 g. We met this objective while maintaining the ability to monitor electrical activity of the stimulator *in vivo* and diminishing complications associated with implantation of the battery pack. The biocompatibility has been optimized to be at least equal to available devices with the external coating matching other implantable apparatuses used in rats^6,7^. While the implantation of the battery board does add a step to the surgery, it is minimally invasive and requires little technical skill to implant. The advantages of this system are coupled to a reasonable cost (~$30.00/unit including batteries) and at least a 30-day life-time. Given that no implanted devices failed within the 30-day testing phase, experimental protocols could be extended considerably. Other advantages of this device include the ability to study the effects of the stimulator in an on/off state under different frequency modes and chronic stimulation. This is of critical importance, as clinical data indicates that some effects of DBS occur immediately, while others require longer durations of stimulation. Finally, the optimization of 2-photon imaging in the surrounding area provides a unique opportunity to observe cellular changes related to electrical brain stimulation. The optimizations performed to allow for concomitant 2-photon imaging could also be compatible with other imaging systems, such as miniscopes^12^, which have the additional advantage of being applicable to freely moving animals.

We further demonstrate that our device can mimic effects previously observed in patients in both WT and transgenic animals. With WT mice, we show that our stimulator induces microglial activation. This is consistent with other animal studies which indicate that valence of change of microglia activation is at least partially dependent on disease status, as healthy animals tend to display increased immune activation, whereas animal models of stroke and Parkinson’s disease are rather characterized by decreased microglial activation. These findings highlight the importance of studying the effects of brain stimulation in animal models where variables such as disease status and stage can be strictly controlled to determine how the consequences of stimulation are regulated by underlying cellular properties. By combining different stimulation parameters with 2-photon imaging in CX3CR1 mice, detailed information regarding changes in microglia morphology could easily be obtained across time. In addition to proximal changes in microglia, we also observed contralateral effects on the expression of GAP-43, a well-established marker of axogenesis. Similar to our results, GAP-43 has previously been shown to be impacted in both ipsi- and contralateral hemispheres after electrical stimulation in rats^13^. While induction of this transcript has not been directly tested in humans, indirect evidence such as reduction of GABA release^14^ and electrical activation^15^ in areas remote from the stimulation site, supports its relevance as GAP43 has been shown to correlate with changes in electrical activity^13^.

In studies using Thy-1 α-Syn mice, we demonstrate motor improvement after 30 days of continuous stimulation. The ability to stimulate constantly, as is done in patient population, is critical as different durations of exposure are known to have different consequences. For example, an almost instantaneous onset of clinical benefit is observed for essential and resting tremors associated with Parkinson’s disease, while a month or more may be required to see improvement in dystonia^16,17^. The overall consistency of our findings with clinical data is of critical importance as brain stimulation has been used as a therapy for different disorders. These applications are only increasing as new technologies-such as specialized electrodes and closed-loop circuits^18,19-^ improve the specificity of targeting and decrease side effects.

While important for clinical DBS research, our novel stimulator also opens up a wide range of research opportunities as exogenous brain stimulation has been shown to influence all aspects of behaviour with effects on obesity^20^, sleep-wake cycle^21^, movement^17^, mood^22^, and cognition^23^ in both pathological and normal conditions^24^. At a cellular level, brain stimulation has been shown to alter inflammation, release of neurotransmitters^25,26^ and differentiation of neuronal precursor cells to neurons and oligodendrocytes^16,27^. However, all techniques have their limitations, and the technology described in this article is no exception. Stimulators are invasive and can alter aspects of brain physiology far from the injection site. In certain circumstances such an activation of large cortical areas may present an advantage for understanding how populations of cells that are regionally connected work together. There are already methods available such as optogenetics and chemogenetics, which can provide the opportunity to activate specific cellular populations. By combining findings from regionally and genetically related cell populations, these techniques will allow for the dissection of the contribution of different cellular populations to behavioural outputs. As such, we feel this newly developed device has the potential to significantly enhance our capacity to understand brain function in health and disease.

## Materials and Methods

### Biphasic micro-stimulator device

The micro-stimulator and battery board were conceptualized and developed in collaboration with Primma technologies (Quebec, Canada) (Fig. 1a). Each unit consists of approximately 30 individual parts micro-soldered onto the circuit boards (Fig. 1b). The weight and dimensions of the micro-stimulator were reduced to allow animals to carry the devices for a prolonged period of time. Power requirements were similarly diminished to reduce the frequency of battery changes. The stimulation parameters were programmed into each micro-stimulator using a micro-controller unit and dedicated external circuit. Parameters could be changed after surgery but a different procedure involving the battery board would need to be utilized in this instance. Each device was additionally equipped with a red LED indicator linked to the power circuit board to notify experimenters of low power. The final weight of the batteries, battery support, board and connectors together was 4.7 g.

Further information on the properties of the micro-stimulator and a detailed description of Fig. 1c are available in the Supplementary Materials and Methods.

#### Stimulation parameters

Frequency was set to ~130 Hz with a balanced biphasic pulse. The current amplitude was 149 μA per pulsation with an average inter-pulse time of 7.78 ms (Fig. 1d). The rationale for the selection of these parameters is explained in detail in Arsenault *et al*.^1^. At the front of the power control board is the flat flex connector (FFC) used to connect the flat cable to the electrodes implanted into the brain. Further information regarding the type of electrodes and a detailed description of Fig. 1e are presented in the Supplementary Materials and Methods. The code for the stimulation parameters was written using the PC Lite-FET Pro430 software from Elprotronic.

### Coating

The micro-stimulators and battery boards were cleaned twice with flux remover for PC Boards (MG Chemicals, Canada, #4140) and a hog hair cleaning brush (MG Chemicals, Canada, # 852) and subsequently dried for 48 h. Once completely dry, micro-stimulators went through 2–3 sequential coating steps to prevent fluid infiltration after *in vivo* implantation.

### *In vitro* validation

The complete micro-stimulator system, including battery boards, micro-stimulators, cables and electrodes, was assembled and placed into two petri dishes (Corning Incorporated, USA, #430165) filled with 0.9% saline solution and covered in parafilm with the connectors and battery boards exposed to the external environment. One petri dish contained the battery board and micro-stimulator, while the other contained the electrodes (Fig. 2a). These dishes were housed in an incubator maintained at 37 °C for 4 weeks. Each week, the voltage output of the micro-stimulator was checked by using a port in the micro-stimulator which was designed to mimic the voltage output of the electrode. This port contained wires which were connected to an external picoscope.

### Animals

Multiple different animal models were used to address the feasibility of different applications of the micro-stimulator. All mice were bred and/or maintained in our in-house breeding facility under standard laboratory conditions (12:12 h dark/light cycle, water and food *ad libitum*, weekly cage cleaning). Mice were group-housed in ventilated cages prior to surgery after which they were individually housed in specialized cages that did not contain any metallic parts to prevent damage to the electrode or micro-stimulator system. Water was provided in glass water bottles (Bio Serv, Canada, #9019) designed to prevent the cable from catching on the sipper tube. All experiments were performed in accordance with the Canadian Guide for the Care and Use of Laboratory Animals, and all procedures were approved by the animal research committee of the Centre de Recherche du CHU de Québec – Université Laval.

#### Electrode implantation

Mice were placed into a stereotaxic frame (David Kopf Instruments, USA, #900) with a mouse gas anaesthesia head holder (David Kopf Instruments, USA, #923-B) and the surgical site was disinfected. After disinfection, the skin on top of the skull was detached and the periosteum was removed. For electrode implantation, one or two holes were drilled into the skull at the site of interest depending on whether a monopolar (subthalamic nucleus stimulation) or bipolar (motor cortex) electrode was utilized. For WT mice, a bipolar electrode was implanted in the M1 region of the right hemisphere motor cortex (anteroposterior −0.2 mm for the back wire of the electrode, mediolateral −1.0 mm, dorsoventral −0.6 mm). For Thy1-αsyn mice, a monopolar electrode was implanted into the subthalamic nucleus of the right hemisphere (anteroposterior −2.0 mm, mediolateral −1.5 mm, dorsoventral −4.4 mm). All stereotaxic coordinates were taken from the mouse brain atlas (Paxinos and Franklin). The accuracy of the surgical implantation of the electrode into the subthalamic nucleus was confirmed by injection of Evan’s blue and post-mortem confirmation of dye location in a number of animals prior to the initiation of the experimental procedures. Deposits of glue (Quick-Bond-Aron Alpha 200, Electron Microscopy Sciences, USA, #72588) were placed at the corner of the open space on the skull and the implanted electrode and its connector were cemented in place with fast curing orthodontic acrylic resin Ortho-Jet TM (Lang dental, USA, #1334) using a 20 G X 1″ needle (Fisher, Canada, #NN-2025R).

#### Micro-stimulator implantation

The micro-stimulator implantation was conducted 1–2 weeks following electrode placement depending on the recovery of the animals after surgery. After pre-operative procedures (supplementary materials and methods) were completed, an incision was made in the upper back between the shoulder blades and a subcutaneous pocket was opened using clamps. A few drops of isotonic saline were applied to the subcutaneous pocket and the micro-stimulator was inserted and centered in the lower back. Two incisions were then made in the skin such that the connectors could be pushed out through these incisions. The apparatus was held in place with two subcutaneous sutures (5-0 coated vicryl Ethicon, Johnson & Johnson, Markham, Ontario, Canada, #J391H) attaching the surgical mesh to the surrounding connective tissue. The incision on the top of the back was closed with mattress sutures (to provide a better distribution of tension) and an antibiotic ointment (PolydermTM, Taro pharmaceuticals Inc, Canada, #02181908) was applied around the connectors and sutures. A flexible cable (Mouser Electronics, USA, #538-15167-0708) was used to connect the micro-stimulator to the electrode. The connections of the cable with the electrode and the board were covered in silicone (Transparent Silicone I, General Electric Company, USA, #SE1124 31623). Finally, the battery board was installed (see summary Fig. 3). Mice were left to recover from surgery and to adapt to the micro-stimulator for one week prior to the start of the protocol.

### Imaging

#### Micro-prism/coverslip assembly

We used right angle micro-prisms with a 1.5 mm square base and aluminum coating on the hypotenuse (Tower Optical, USA, part 4531-0023) to maximize light reflection in the NIR range. We had 5 mm diameter coverslips drilled (Potomac Photonics, USA) such that both electrodes could be inserted into two 0.3 mm diameter holes spaced 1.2 mm apart in the coverslip. Micro-prisms were fixed on coverslips using Norland optical adhesive and UV light such that the flat surface perpendicular to the coverslip was facing the electrodes, thereby allowing sideways imaging around the insertion site.

#### 2-photon imaging

Prior to imaging, mice were anaesthetized and maintained as previously described. After induction, the head of the mouse was firmly fixed and positioned under the microscope objective using the head plate and holding bar. A fluorescent vascular tracer (70 kDa Dextran-TxRed) was injected i.v. to label blood vessels. Imaging was performed using a high NA, 25X magnification objective (XLPLN25XWMP2, Olympus) on a FVMPE-RS system (Olympus) using FluoView software. Image processing was performed with Fiji (NIH, USA).

### Statistical analysis

Statistical analyses of body weight were performed using a repeated measures One-Way ANOVA with Bartlett’s post-tests. Normality was confirmed using the D’agostino and Pearson omnibus normality test and equal variance was confirmed using Bartlett’s tests of variance. Statistical analysis of behavioral data after stimulation was completed using a Two-Way ANOVA followed by Fisher’s LSD test.

## Supplementary information

Supplementary Materials and Methods

Supplementary Video 1

## Data Availability

The datasets generated during the current study are available from the corresponding author upon reasonable request.
