# Supplementary material for: A novel wireless brain stimulation device for long-term use in freely moving mice
Source: Sci Rep. 2019 Apr 23;9:6444. doi: 10.1038/s41598-019-42910-7 (PMC6478908; doi:10.1038/s41598-019-42910-7)
Supplement: Supplementary file 1 — Supplementary Materials and Methods [file 41598_2019_42910_MOESM1_ESM.docx]

**A novel wireless brain stimulation device for long-term use in freely moving mice**

Melanie Alpaugh, Martine Saint-Pierre, Marilyn Dubois, Benoit Aubé, Dany Arsenault, Jasna Kriz,

Antonio Cicchetti, Francesca Cicchetti

**SUPPLEMENTARY MATERIALS AND METHODS**

**Biphasic micro-stimulator device**

*Micro-stimulator parameters:* Power for the micro-stimulator came from two 393-type batteries with a voltage of 1.55 V each, mounted on the upper part of the micro-stimulator to provide a total of 3 V to power the circuit (**Figure 1a**).

*Electrical parameters:* The design of our micro-stimulator prototype is based on the development of a portable external biphasic current generator for which the electronic circuit is illustrated in **Figure 1c**. The modulation of the electric pulse frequency and duration is insured through a micro-controller (MSP430G2230IDR; Texas Instruments, USA). The first analog switch (SN74LVC1G3157DRLR, Texas instruments) outputs 0 V, when both micro-controller signals are at a low level and 1.8 V, when the micro-controller signals are at a high level. The output of this switch is then halved to 0 or 0.9 V by two 10 KΩ resistors which are connected to the positive input of the operational amplifier. Following operational amplifier theory, the voltage applied to the positive input of the operational amplifier is equal to the voltage present on the negative input. The operational amplifier is configured as a current source with an output current value of 0 μA or 0.9 V / Resistance value = (0.9 V) / (6040 Ω) = 149 μA. The 0.9 V value was chosen to optimize the operational amplifier dynamic output voltage for the current regulator. The output voltage margin is 2.1 V, which maximizes the impedance range in which the current can be regulated into the mouse brain. The mouse brain has a complex impedance with unknown resistance, capacitance and inductance values. However, the micro-stimulator outputs 149 μA whenever the impedance connected to both electrodes is below approximately 20 KΩ. The voltage waveform is not a square wave and cannot exceed 2.1 V peak (saturated operational amplifier output). The operational amplifier output voltage is stabilized by one 1 MΩ resistor and one 100 pF capacitor. Furthermore, a Schottky diode (NSR0240P2T5G, ON semiconductor) prevents output glitches and helps provide dead time - where the current source is zero - between the positive and negative current pulses. Two more analog switches (SN74LVC1G3157DRLR, Texas instruments), also under the control of the micro-controller, form an analog H-bridge switch which allows injection of positive or negative 149 μA current pulses into the mouse’s brain. The first analog switch is further used to allow dead time. A low-dropout voltage reference (REF3318AIDCKT, Texas Instruments, USA) is used to convert 3 V battery voltage to 1.8 V in order to ensure precise pulsed current injection into the mouse’s brain. The battery voltage is monitored by a low voltage detector (TPS3801-01, Texas Instruments, USA). When the battery voltage reaches 2.28 V, a low power LED oscillator is activated, triggering blinking of an external red LED (SML-P11VTT86, Rohm Semiconductor, Japan). A P-channel MOSFET (RZM001P02T2L, Rohm Semiconductor, Japan) in series with both batteries is used as an ideal diode (minimizing voltage drop) and provides protection against reverse battery polarity.

**Coating**

For the first step, dry micro-stimulators were embedded either in 1 layer of epoxy for 5 min (Adhaero, China) and dried for 24 h or 3 layers of aerosolized silicone conformal coating (MG Chemicals, Canada, #422B) applied at 5-min intervals and dried for 48 h. For the second step, micro-stimulators were embedded in 1 layer of epoxy for 5 min and dried for 24 h, 3 layers of aerosolized silicone applied at 5-min intervals and dried for 48 h, or 2 layers of gel silicone (Dow Corning, USA, #MIL-A-46146) applied at 15-min intervals and dried for 48 h. For the third step, micro-stimulators were coated in 2 layers of gel silicone. Each treatment was alternately applied to each side of the micro-stimulator. The exact combinations are summarised in **Figure 1c**. After cleaning and drying, the base of the battery boards were embedded in 1 layer of epoxy (Adhaero, China) and dried for 24 h. The top of the battery board was not in contact with the animal and was, therefore, not coated.

**Electrodes**

We developed three types of home-made electrodes to facilitate stimulation of both surface and deep brain structures for multiple applications.

*Bipolar electrodes for brain stimulation of surface structures:* Two stainless steel wires (Bare: 0.005" or Coated: 0.008", A-M systems, USA, #7916000) of 7 and 8 mm in length were stripped starting 0.25 mm from each tip. Each wire was bent to an angle of 90°, 2 mm from the tip to form the portion implanted into the brain. The opposite end of each wire was also stripped and welded onto one prong of the connector (2 position receptacle connector through hole gold, Digi-key, Electronics, USA, #3M9397-ND). Two different wire positions were developed, one where the wires were welded to the prong at a 90° angle (vertical position, **Figure 1e**) and one in which the wires were welded parallel to the prong (horizontal position, **Figure 1e’**). All components around the prongs were coated in a layer of epoxy (Adhaero, China) to hold everything in place. All electrodes were tested in saline solution (Hospira Healthcare Corporation, Canada) to ensure proper functioning prior to use. Bipolar electrodes were selected due to the greater regional specificity generated as compared to monopolar electrodes [^1^](#_ENREF_1).

*Monopolar electrode for brain stimulation of deep structures:* A 30 G X 1/2 precision glide needle (BD, Canada, #305106) was cut at both ends to obtain a 7 mm stainless steel tube. We then inserted a Platinum-iridium wire (Bare: 0.002", Coated: 0.004", A-M systems, USA, #776000) that had been stripped 0.25 mm from the tip inside the needle tube, keeping only a small length of coated wire outside of the tube. The tube was welded to one prong of the connector using acid paste (soudotec, Boc-Gaz FOG1 #402186001) and a soldering iron. The other end of the wire was also stripped where it would connect with the second prong, the wire was pulled through the tube, wrapped and welded around the second prong of the connector. All components around the prongs were coated in a layer of epoxy (Adhaero, China) to hold everything in place (**Figure 1e’’**). All electrodes were tested in saline solution to ensure proper functioning prior to use. Monopolar electrodes were selected for stimulation of deep brain structures in order to avoid the greater tissue damage associated with implantation of a bipolar electrode [^1^](#_ENREF_1). This approach has recently been validated in a rat study where effects of subthalamic nucleus stimulation with bipolar and monopolar electrodes were compared, and monopolar were found to be more effective [^2^](#_ENREF_2).

**Animals**

*Wild Type (WT) C57BL/6* mice underwent surgery between 5.5 and 7 months of age to determine tolerability of the implanted micro-stimulator. *Thy1-αsynuclein (Thy1-αsyn)* mice, a model of Parkinson’s disease displaying a progressive motor phenotype due to overexpression of human WT α-syn under control of the mouse Thy1 promoter, underwent surgery between 5.5 and 7 months of age and were assessed for their behavioral responses following chronic stimulation of the subthalamic nucleus. *CX3CR1-GFP* and *C57Bl/6-Tg(Gap43-luc/gfp) 10 Kri* mice underwent surgery at 6 and 2 months of age respectively, for studies involving imaging. *Cx3CR1-GFP* mice were used for 2-photon imaging as it permitted visualization of microglia in live animals through the expression of GFP in Cx3CR1 positive cells. *C57Bl/6-Tg(Gap43-luc/gfp) 10 Kri* mice were used for *in vivo* bioluminescence imaging (not shown here) and post-mortem studies to identify cells that are expressing Gap43, a marker of axogenesis.

*Imaging studies: CX3CR1-GFP* male mice underwent an optimized surgical procedure for intravital 2-photon imaging while transgenic *C57Bl/6-Tg(Gap43-luc/gfp) 10 Kri* mice [^3^](#_ENREF_3) were used for immunohistochemistry and *in situ* hybridization experiments.

*Pilot implantation studies: C57BL/6* male mice underwent surgical implantation of the electrode to monitor health status. After a two-week recovery period, a subset of these mice underwent surgical implantation of the micro-stimulator.

*Treatment studies:* *Thy1-αsyn* male mice maintained on a C57BL/6J background underwent surgical implantation of the micro-stimulator and electrode for the purpose of performing behavioural studies and determining whether our apparatus could replicate clinical work performed in Parkinson’s disease patients receiving DBS.

*Anaesthetic and preparative procedures:* Anaesthesia was induced using 3-4% isoflurane (Corporation AbbVie, Canada, #1000006291) in oxygen (0.8-1.5L/min) in an induction chamber. Surgical plane was confirmed by checking the plantar reflex. Mice were maintained under 1.5-2% isoflurane in oxygen (0.5L/min) and the depth of anaesthesia was monitored by observing breathing rate. While under anaesthetic, body temperature was maintained using a Gaymar T/pump (Gaymar Indutries Inc, USA, #TP-400) and pads (Gaymar Indutries Inc, USA, #TP-3E). Immediately after induction, the mouse received a subcutaneous injection of 0.5 mL of saline solution, 0.1 mL of lidocaine/bupivacaine (lidocaine- Astra Zeneca, Canada, #021; Bupicacaine -2.5 mg/ml Hospira Healthcare Corporation, Canada, #01559) and 0.03 mL of 0.5-1 mg/kg buprenorphine slow release (Chiron, USA) and were prepared for surgery by shaving the head (Harvard Apparatus Canada, #72-9063) and applying ophthalmic ointment to the eyes (Refresh Lacri-Lube, Allergan Inc, Canada).

*Micro-stimulator implantation:* Prior to implantation, the micro-stimulators were cleaned with 70% ethanol (Commercial Alcohols, Brampton, Ontario, Canada), rinsed in saline solution, and wrapped in sterile polyester mesh (SurgicalMesh, USA, #PETKM2006) held in place with sutures (using 5-0 coated vicryl Ethicon, Johnson & Johnson, Canada). Finally, the connectors of the micro-stimulator were wrapped in parafilm (Fisher, Canada, #PM-992) to prevent infiltration of biological fluids during the surgical procedure.

*Voltage and pulse measurement:* Each week, voltage and amperage were measured to ensure proper functioning. In order to access the battery board, mice were anaesthetized prior to the start of the procedure. Once under anaesthetic, the battery board was removed and temporarily replaced with a board containing wires connected to the C (com_lead), V (volt) and S (shunt) ports. To measure voltage, an external oscilloscope was connected to the C and V wires, while current was measured by connecting the oscilloscope to the C and S wires to obtain a voltage which could be used to calculate amperage by dividing the value by the resistance of the wire in the port (499 Ω) according to ohms law. The electrical measurements were processed using the program PicoScope6 (PC Oscilloscope software, version 6.8.8.16, Pico Technology Ldt). Following the confirmation of appropriate functioning, the batteries were replaced each week to ensure sufficient power was supplied to the apparatus at all times, although the anticipated life of the batteries was two weeks.

*Post-surgical monitoring:* Mice were observed daily and weighed throughout the post-operative period as well as during the brain stimulation protocol. Throughout the experiment, mice received a high calorie gel and soft food. If any rash or injury appeared after surgery, mice were topically administered green clay after disinfection with chlorohexidine.

*Behaviour:* After surgery, but prior to the start of stimulation, mice were trained on the *narrow beam* for two consecutive days prior to baseline testing. On these testing days, mice were placed at the wide end of the narrow beam and left to cross to the dark enclosure situated at the narrower end of the beam. Mice that fell were immediately replaced on the beam at the point at which they fell. Mice underwent three consecutive trials and were recorded during testing with a mirror opposite to the camera to permit observation of errors on both sides of the beam. Videos were scored for time to cross and errors (foot slips and falls) by a blind observer. A partial foot slip incurred a score of 1, a complete slip a score of 2, and a fall as score of 3. For *open field* analysis, mice were tested individually for 60 min in a PAS-home cage system consisting of a square Plexiglas arena (16” X 16”) equipped with 25 X 25 photobeams that records beam breaks in real time. The distance travelled, average speed, as well as fine and ambulatory movements in the center of the field and the periphery were retrieved from the PAS software in 5-min bins. This data was used to measure locomotor activity and anxiety-related behavior. For all testing, mice were allowed one hour to acclimate to the testing room before starting.

*Cranial window:* Preparation for surgery and stereotaxic frame use were as described above. Subsequently, a square window was created in the skull using a micro-drill (Harvard Apparatus Canada). If bone dust began to accumulate, it was removed from the skull surface with a saline solution applied by a cotton swab. Once the brain was exposed, the dura was removed using small sharpened forceps with particular care to avoid damage to the surrounding blood vessels. Superficial bleeding was controlled using a small piece of sterile surgical foam. The microprism/coverslip assembly was carefully inserted in the cranial window to avoid blood vessels and then the edges were sealed with glue. Once the latter was dry, the head plate was positioned and fixed with dental cement (Lang Dental, Wheeling, IL, USA) and electrodes were then inserted into the brain through the hole on the coverslip. Head plates were custom made (Acier inoxydable Den-Mar, Qc, Canada) out of stainless steel and weighed less than 0.4 g. Finally, the cranial window and surrounding skull area were covered with dental cement. The whole procedure, from anaesthesia to sealing with dental cement, took less than 30 min.

**Post-mortem analyses**

*Immunohistochemistry for neuronal and microglial elements:* Sections were washed in phosphate buffer saline (PBS) 0.2M pH 7.4 and placed for 30 min at room temperature (RT) in a 3% hydrogen peroxide to eliminate endogenous peroxidase activity. The sections were then washed and incubated in a blocking solution containing 4% normal goat serum (NGS; Wisent Bioproducts, St-Bruno. QC), 4% Triton-X100 (Sigma, ON, Canada) and 1% bovine serum albumin (BSA; BioShop Inc, Canada) in PBS 0.2M. After overnight incubation at 4°C with an antibody against Iba-1 (Wako Chemicals, Richmond, VA; 1:1000), the sections were extensively washed in PBS and incubated for 1 h at RT in a PBS solution containing biotinylated goat anti-rabbit IgG (Vector Labs, Burlington, ON; dilution 1:1500), Triton X-100 (0.2%) and BSA (1%). After further washing in PBS, the sections were placed in a solution containing avidin-biotin peroxidase complex (ABC) (Elite kit; Vector Labs, Burlington, ON) for 1 h at RT. The bound peroxidase was revealed with nickel-intensified 3,3’-diaminobenzidine tetrahydrochloride (DAB; Sigma-Aldrich, ON, Canada) as the chromogen. After immunostaining for Iba-1, the sections were re-incubated overnight at 4°C with an antibody raised against NeuN (Millipore, Temecula, CA; 1:2500). The incubation procedures were the same as above, except that the incubation with the secondary antibody was with a goat anti-mouse antibody (Vector labs, Burlington, ON; 1:1500) and sections were visualised using 0.05% DAB and 0.01% hydrogen peroxide in 0.05 M Tris-imidazole (pH 7.2) at RT. The reaction was stopped by extensive washing in PBS. Sections were mounted on Superfrost slides (Fisher Scientific, ON, Canada), dehydrated in ascending baths of ethanol, delipidated in citrisolv (Fisher Scientific, ON, Canada) and coverslipped using DPX mounting media (Electron Microscopy Sciences, Hatfield, PA).

*In situ hybridization for GAP-43 expressed in axonal growth:* Brain sections were washed with autoclaved PBS 0.1M pH 7.4 containing diethylpyrocarbonate (PBS/DEPC) and incubated in hydrogen peroxide 3% for 30 min at RT. Sections were washed in PBS/DEPC and incubated in a filtered blocking solution with 1% BSA, 0.1% Triton X-100 and PBS/DEPC. After overnight incubation with mouse anti-NeuN (1:2500) diluted in filtered blocking solution with 2.5 mg/mL of heparin sodium salt (MP Biomedical), sections were washed and secondary incubation, visualization and mounting were performed as described above in RNase free conditions. After mounting, slides were air-dried for 1 h. Pre-hybridization was performed on the same day in RNase free conditions. A specific [^35^S]UTP-labeled complementary RNA (cRNA) probe was used to assess tissue mRNA levels of GAP43 in the NeuN stained sections. The cRNA probe for GAP43 was cloned into a pBluescript II SK+ plasmid. Linearization was made with the BAMH1 enzyme. The antisense probe was synthesized with [^35^S]UTP and T7 RNA polymerase. Brain sections were fixed in 4% PFA pH 9.5 at RT for 5 h. Pre-treatment involved the use of consecutive incubations (PBS 0.1 M twice 5 min, proteinase K 0.1 μg/ml 25 min at 37 °C, acetylation bath (0.25% acetic anhydride, triethanolamine 0.1 M) 10 min, 5 min in standard saline citrate (SSC) (0.3 MNaCl, 30 mM sodium citrate)). Successive baths of ethanol solutions (30%, 60%, 100%, and 100%; 10 dips) were used for dehydration. *In situ* hybridization of the riboprobes on tissue sections was performed at 58°C overnight in a standard hybridization buffer (deionised formamide 50%, sodium chloride 5 M, Tris 1 M, EDTA 0.5 M, Denhart's solution 50X, dextran sulfate 50%, tRNA 10 mg/ml, DTT 1 M, and 35S coupled 10^6^ cpm/μl probe). Post-treatment was conducted using different successive baths: SSC 4X (30 min), removing coverslips, SSC 4X with DTT 1M four times (5 min), RNase A 20 μg/ml (30 min) at 37 °C, SSC 2X with DTT 1M twice (5 min), SSC 1X with DTT 1M (5 min), SSC 0.5X with DTT 1M (10 min) and SSC 0.1X (30 min) at 60°C. Repetitive baths of ethanol solutions (50%, 70%, 95% and 100%; 10 dips) were used for further dehydration. Tissue sections were then placed against BiomaxMR (Kodak, New Haven, CT) radioactive sensitive films. Autoradiograms were developed following a 48 h exposure. Argentic emulsion was further added to allow for visualization under the microscope. Delipidation was performed with 4 baths of ethanol, 2 baths of Citrisolv and 3 baths of ethanol. Following these steps, slides were dipped in NTB emulsion (VWR, ON, Canada) melted at 42°C, air-dried for 4 h and stored in the dark for 7 days at 4°C. The emulsion was then developed (3.5 min) in a D-19 developer (Kodak), rinsed in deionised water and fixed (5 min) in Rapid Fixer solution from Kodak. Slides were rinsed in deionised water for 1 h followed by ethanol dips and 3 Citrisolv baths (2 min). Slides were coverslipped with DPX mounting media.

**References**

1 Chopra, A. *et al.* Underlying neurobiology and clinical correlates of mania status after subthalamic nucleus deep brain stimulation in Parkinson's disease: a review of the literature. *The Journal of neuropsychiatry and clinical neurosciences* **24**, 102-110, doi:10.1176/appi.neuropsych.10070109 (2012).

2 Badstuebner, K., Gimsa, U., Weber, I., Tuchscherer, A. & Gimsa, J. Deep Brain Stimulation of Hemiparkinsonian Rats with Unipolar and Bipolar Electrodes for up to 6 Weeks: Behavioral Testing of Freely Moving Animals. *Parkinson's disease* **2017**, 5693589, doi:10.1155/2017/5693589 (2017).

3 Gorup, D. *et al.* Increased expression and colocalization of GAP43 and CASP3 after brain ischemic lesion in mouse. *Neuroscience letters* **597**, 176-182, doi:10.1016/j.neulet.2015.04.042 (2015).

**SUPPLEMENTARY FIGURE LEGENDS**

**Supplementary video 1:** Time-lapse video of microglia movement in a Cx3CR1-GFP mouse concomitant with electrode stimulation.
